# Diet in Disease

**Published:** 1892-12-31

**Authors:** Ernest Hart

**Affiliations:** Bachelier-ès-Sciences-es-Lettres (rèstreint), formerly Student of the Faculty of Medicine of Paris, and of the London School of Medicine for Women


### Diet in Disease.

By Mrs. Ernest Hart, Bachelier-ès-Sciences-es-Lettres (rêstreint), formerly Student of the Faculty of Medicine of Paris, and of the London School of Medicine for Women.

### XI.—INVALID FOODS.

The preparation of food for those who are seriously ill is a matter of vital importance, for the life of the patient often depends either on the maintenance of strength during the acute period of the disease or the recovery of strength during convalescence. In acute illness and in high fever the stomach is unable to digest solid food. It becomes, therefore, of great importance to administer food which is not only highly nutritious, but which contains the food principles necessary for the maintenance of strength and the repair of the tissue wasted in the fever process. It is only of recent years, however, that the feeding of the patient has been based on scientific principles, and that doctors have turned their minds to such subjects as the correct making of beef-tea and gruel. Even now, unfortunately, the provision of food for the very rich is entrusted too much to the vendors of patented and secret preparations, and we are left in ignorance of the actual constitution of the foods for which we are paying a high price, in the hope that they contain the elements necessary. In this we may be, however, entirely deceived, and many of the patented beef-teas and meat-juices which are purchased at great cost, in the belief that they are "strengthening," contain only a trace of albumen. The expensive preparations of malt also advertised as "foods" cannot be properly included in this category.

My object will be to show how the most nutritious invalid foods can be prepared at home, in the sick-room, and at the least cost. I have, when attending on the sick, been frequently struck by one of two things—either the immense cost at which the patient was being nourished on patent foods, or the small amount of nourishment which was extracted by means of ignorant methods from good materials.

If the nature of the food principles necessary for the maintenance of the body be remembered, and also the broad facts of digestion, beef-tea, jellies, &c., would be made with much more intelligence, and the invalid would be better fed. Having, in previous papers, given some account of food values and of the processes of digestion, I will proceed to describe how the invalid may be intelligently fed, without resorting to costly patented foods of unknown composition, and will give the recipes which may be safely followed.

### Beef-Teas and Beef-Juices.

**Beef-Tea.**—*Methods of Making.*—1. Remove all the fat and skin from one pound of fresh gravy beef; cut it up in small pieces, and put it in a stone jar with a pint of cold water and a little salt. Replace the lid of the jar, and let it stand all night. The next morning place the jar in a saucepan of boiling water, and let it simmer gently, but never boil, for five hours. Strain the fluid from the beef through a colander.

It must be borne in mind that beef tea made in this way is, as well as the patent beef-teas and beef-juices, not a food in the true sense of the word, but rather a stimulant. Such beef-teas contains little or no albumen, and only a very slight amount of gelatine, but they hold in solution the sapid extractives and salines of the meat. The universal experience, however, is that beef-tea is to the sick and weak a valuable restorative, though it must never be forgotten that it is not nourishing.

2. *Whole Beef-Tea.*—Make the beef-tea as in the previous case, but instead of throwing away the residue of the meat, pound it in a mortar into a pulp, pass it through a wire sieve and add it to the beef-tea. The beef-tea made by this method is thoroughly nutritious, as all the fibre and albumen of the meat are contained in it.

3. *Peptonised Beef-Tea* (Sir William Roberts' recipe).—Mix half a pound of finely minced lean beef with half a pint of water and 20 grains of bicarbonate of sodium. Let it simmer for an hour. Remove it from the fire, and when it has cooled down to a lukewarm temperature add a tablespoonful of *liquor pancreaticus.** Then set the mixture aside for three hours, wrapped in a tea cosy or flannel to maintain the temperature, and occasionally shake it. At the end of this time decant the liquid part and boil it for a few seconds.

Beef-tea prepared in this way is as rich in albuminates as milk. When seasoned with salt it is scarcely distinguishable in taste from ordinary beef tea. By being partly predigested it is eminently suitable for invalids whose digestive organs are in a much weakened condition. Care should, however, be taken not to continue the use of predigested foods too long after the stomach has begun to recover tone, else the stomach becomes demoralised, and may lose the power of normal digestion.

4. *Raw Meat-Juice* (Dr. Cheadle's recipe).—To one part of the best rump steak finely minced add one-fourth the amount of cold water. Stir well together, and allow the beef to soak for half an hour, then place the whole in a piece of muslin or cambric, and forcibly express all the juice by firm twisting.

By this method a highly nutritious and nitrogenous food is obtained, containing no less than five per cent. of albumen. In Dr. Cheadle's opinion raw meat-juice is the most easily digested and restorative of all animal foods, and the most valuable of all nitrogenous preparations for children.

5. *Beef Balls Raw.*—Scrape with a knife all the juice out of a fresh rump steak, leaving nothing but the fibrous tissue behind. Mix with cream and roll into balls. Heat a baking tin very hot, and roll the balls rapidly over the hot surface. Sometimes a drop of cherry brandy is added to each ball to mask the flavour; but I have found that rolling the balls over a hot tin and the addition of cream will take away both their objectionable appearance and raw flavour, while the condition of rawness remains really unaltered. This is a very valuable food in acute gastritis; also in gastric catarrh, when solid food is ill tolerated.

* Liquor Pancreaticus is made from beef pancreas. Pancreatin has the power of digesting albumen and turning it into soluble albumose, like pepsin. The preparations of pancreatin, are much more reliable than those of pepsin, and are therefore used for predigesting or peptonising foods.

### Malted Foods.

We have seen that in the digestion of starch it is acted upon by a diastase which is contained either in the saliva or the pancreatic juice, which diastase converts starch into glucose or grape sugar. Malt has the same effect on the starch contained in wheaten and other meals, at a certain heat. Before being converted into glucose, the starch is first changed into dextrine, then into maltose, and finally into grape sugar. In malted foods, the malt flour is mixed with the finest wheaten flour, and the process of conversion into sugar is started and then stopped. On mixing the malted food with water the process recommences, and is carried on rapidly, either while being cooked or in the stomach, and in a short time the whole of the starch is turned into grape sugar and is ready for absorption. In most of the patented malt extracts sold the change of starch into sugar has been carried very far, and the maltine has, as a food, little greater value than treacle or syrup. Both Sir William Roberts and Dr. Cheadle are agreed that these "malted foods" are quite unsatisfactory as foods if taken only mixed with water; but that, provided they still contain a considerable amount of active diastase, they are, if mixed with milk or gruel, valuable and highly digestible food for invalids and delicate children.

**How to Make Malt Infusion** (Sir William Roberts' method).—Mix three ounces of crushed malt thoroughly well with half a pint of cold water in a jug. Let the mixture stand over night. The supernatant liquid is then carefully decanted off from the sediment and strained through two or three folds of muslin, until it comes through fairly clear and bright. Malt infusion thus prepared has a light brown colour like sherry, a faint maltish taste, and the odour of beer-wort. It is prone to fermentation, and should be prepared fresh every day.

This method of preparing malt infusion is so simple, and the product is so efficacious in aiding the digestion of gruel and farinaceous foods, that it should be regarded as a household remedy. It costs three farthings a pint.

**Malted Gruel.**—The gruel should be well boiled and strained to separate the lumps. When cool enough to swallow, the malt infusion is added. One tablespoonful will digest half a pint of gruel. The action is very rapid; in a few minutes the gruel becomes thin from the conversion of the starch into maltose (Roberts). Other farinaceous foods, such as arrowroot, can be malted in the same way.

### Peptonised Foods.

In cases of extreme debility of the digestive organs or arrest of the digestive function in the stomach, the opportunity which science gives the invalid of having digestion accomplished for him outside of the body is one which the tormented dyspeptic may be thought to avail himself of with eagerness. But the object of using peptonised foods should be always to tide over a difficult time, not to encourage a habit; to give the digestive organs physiological rest, so that they may recover power, not to enervate them by continued disuse. Peptonised foods should therefore be used with caution and under medical advice.

**Peptonised Milk.**—Dilute a pint of milk with a quarter of a pint of water, and heat to a temperature of 140 degrees. Then mix with the hot milk two tea-spoonfuls of liquor pancreaticus and 20 grains of bicarbonate of sodium. The mixture is then poured into a covered jar and placed in a warm place to keep up the heat. At the end of an hour and a half the milk is raised to the boiling point for a few seconds, after which it can be used as ordinary milk.

**The Cold Method of Preparing Peptonised Milk.**—Add half a pint of water and 20 grains of bicarbonate of sodium to a pint of milk, and three tea-spoonfuls of liquor pancreaticus. The mixture is then set aside in a room at about 60 or 65 of temperature for three or four hours, at the end of which time it is ready for use. If used at once it need not be boiled, but if the milk has to be kept any time it is better to bring it to the boiling point for a few seconds so as to arrest fermentation, and to prevent the production of a bitter flavour.

**Peptonised Soups, Jellies, and Blanc-manges.**—These can be prepared with a little ingenuity, it being always borne in mind that the peptonised fluid added to the stock, cream, isinglass, &c., used must have been boiled, and the action of the ferment arrested; otherwise a disagreeable bitter flavour will be communicated to the food, and the result will not be successful. In soups, peptonised gruel can be used instead of water; in jellies it can be added to the isinglass or gelatine and flavouring matters; in blanc-manges peptonised milk is added to cream.

**Peptonised Milk Gruel.**—Make a good thick gruel. While still hot add an equal quantity of cold milk. To a pint of this mixture add two teaspoonfuls of liquor pancreaticus and 20 grains of bicarbonate of sodium. Set aside in a warm place for two or three hours, then raise to the boiling point and strain. The mixture should be watched and tasted from time to time, and boiled as soon as a slight flavour of bitterness is perceived. If the peptonised process is allowed to go too far, the bitterness produced makes the gruel unpalatable.

Peptonised beef tea has been described before.

